# Supplementary material for: The Novel Cerato-Platanin-Like Protein FocCP1 from Fusarium oxysporum Triggers an Immune Response in Plants
Source: Int J Mol Sci. 2019 Jun 11;20(11):2849. doi: 10.3390/ijms20112849 (PMC6600160; doi:10.3390/ijms20112849)
Supplement: Supplementary file 1 [file ijms-20-02849-s001.pdf]

Supplementary Materials:

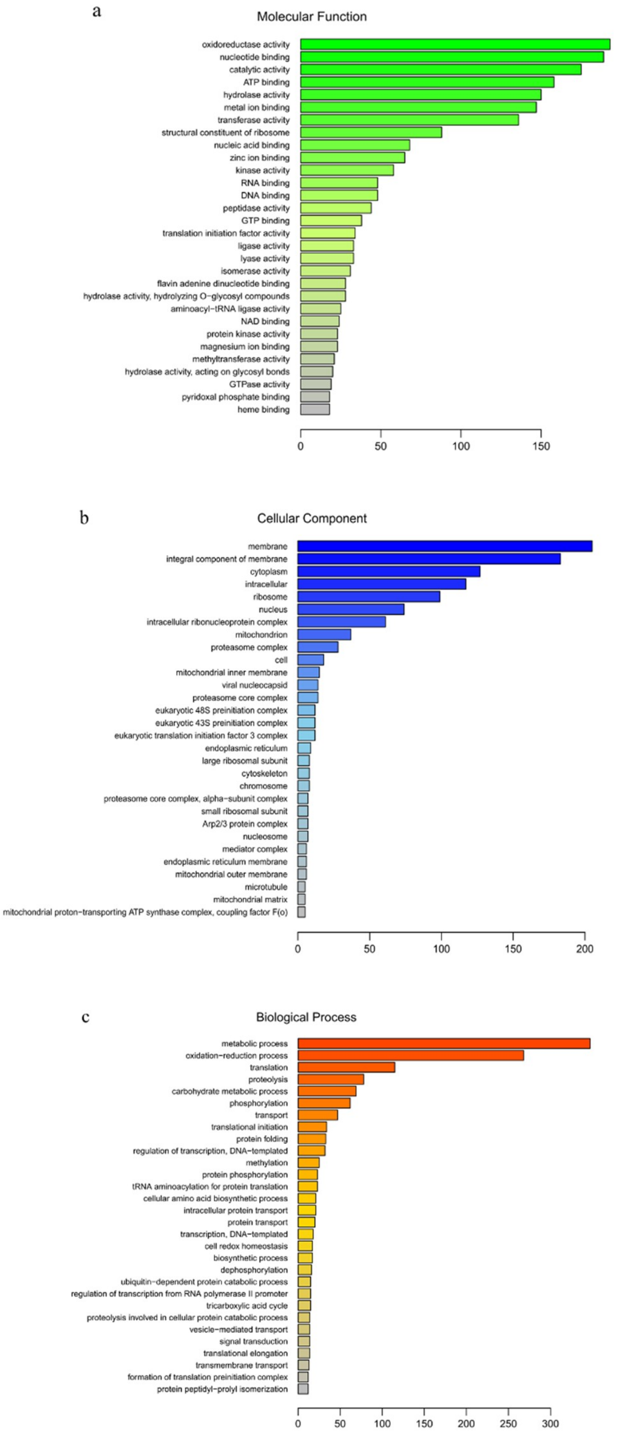

Figure S1: The top 30 identified proteins were categorized with GO enrichment analysis. **(a)** Molecular functions mainly included oxidoreductase activity, nucleotide binding, catalytic activity, ATP binding, hydrolase activity, metal ion binding, and transferase activity. **(b)** Cellular components mainly included membrane, integral component of membrane, cytoplasm, nucleus, intracellular, ribosome, proteasome complex, cytoplasm, and mitochondrion. **(c)** Biological processes mainly included metabolic process, oxidation-reduction process, transport, proteolysis, phosphorylation, carbohydrate metabolic process, translational initiation, proteolysis, translation, and protein folding.

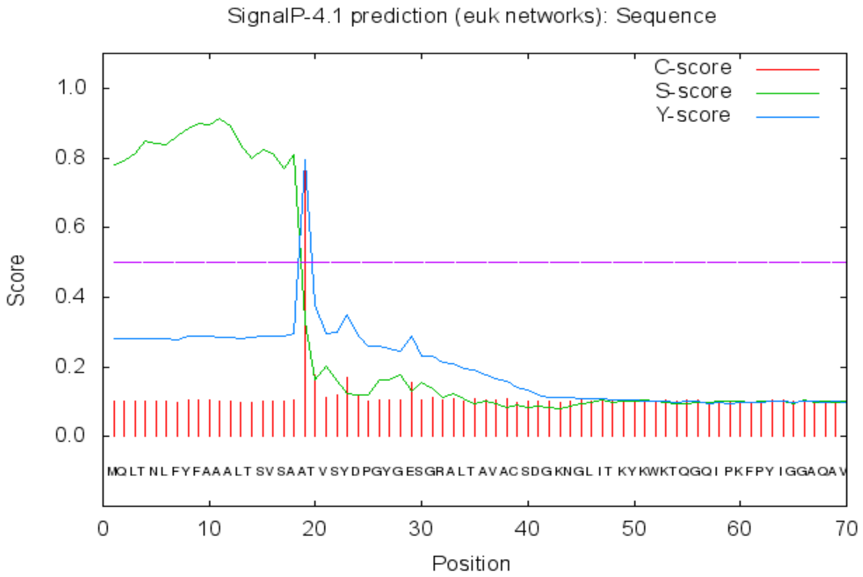

Figure S2: The signal peptide of FocCP1 protein was predicted with SignalP-4.1. The 1-18 amino acid sequence was a signal peptide of FocCP1. C-score and Y-score reached the maximum values of 0.761 and 0.794 at 19 amino acid positions respectively. S-score reached the maximum value of 0.914 at 11 amino acid positions.

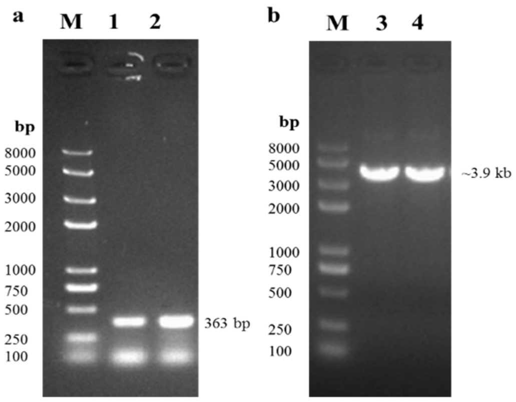

Figure S3: Gel electrophoresis of the *FocCP1* gene and recombinant *pPICZαA-FocCP1* plasmid. **(a)** The full length *FocCP1* gene lacking signal peptides and stop codon was amplified by *pPICZαA-FocCP1*-F/*pPICZαA-FocCP1*-R primers; the open reading frame of the *FocCP1* gene contained a 363 bp nucleotide sequence. **(b)** The recombinant *pPICZαA-FocCP1* plasmid was constructed and reached approximately 3.9 kb in nucleotide sequence length. M: marker, 1 and 2: *FocCP1* gene, 3 and 4: recombinant *pPICZαA-FocCP1* plasmid.

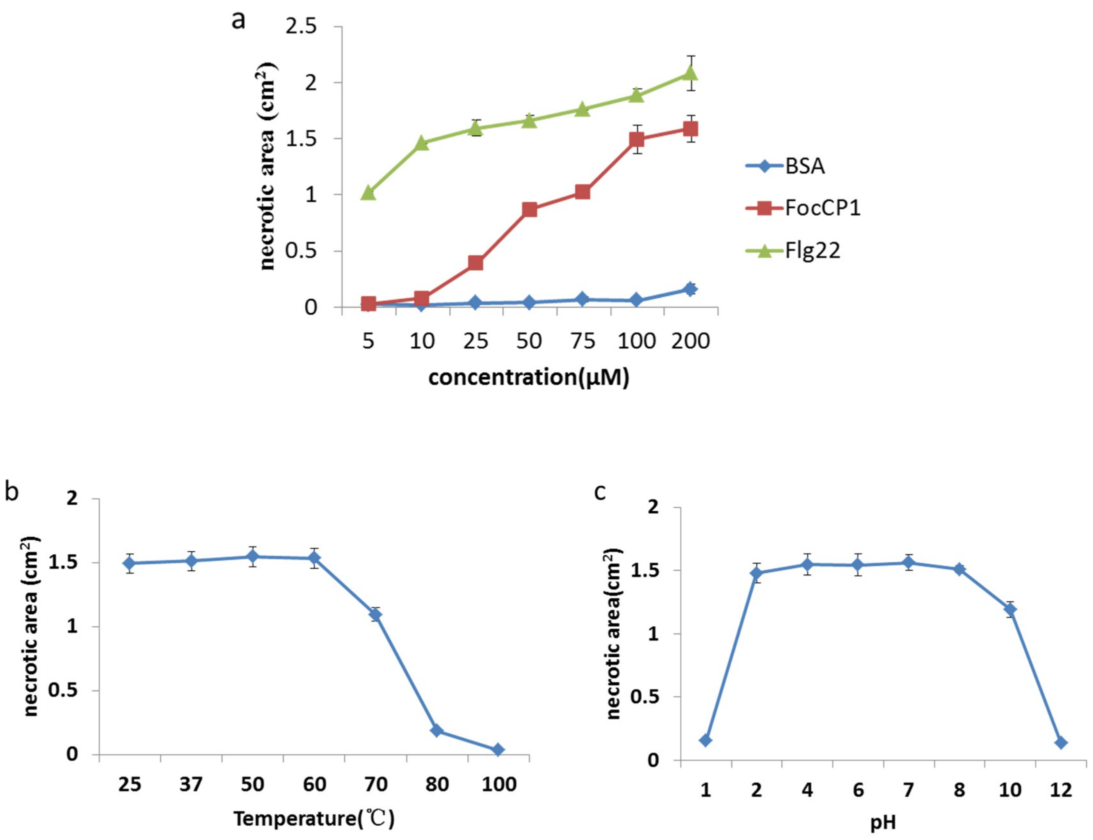

Figure S4: The minimum concentration elicitor function and stability of FocCP1 protein were analyzed. **(a)** The minimum concentrations of FocCP1 were determined for inducing HR in tobacco leaves: 25 μM FocCP1 induced slight HR, 75 μM FocCP1 induced HR, greater than 100 μM FocCP1 induced obvious HR in tobacco leaves. **(b)** The heat stability of FocCP1 was analyzed after treatment with different temperatures. FocCP1 had heat stable below 70°C, while FocCP1 lost elicitor function above 80°C. **(c)** The acid-base stability of FocCP1 was analyzed after treatment with different acidic and alkaline solutions. FocCP1 had acid-base stability within pH 2-10, while FocCP1 lost elicitor function below pH 2 or above pH 10. In all experiments, FocCP1 was infiltrated into tobacco leaves with approximately 2 cm^2 area. Three independent replicates were performed. Values were means ±SE.
